# Long COVID occurrence in COVID-19 survivors

**DOI:** 10.1038/s41598-022-10051-z

**Published:** 2022-04-11

**Authors:** Aya Sugiyama, Kei Miwata, Yoshihiro Kitahara, Mafumi Okimoto, Kanon Abe, Bunthen E, Serge Ouoba, Tomoyuki Akita, Naoki Tanimine, Hideki Ohdan, Tatsuhiko Kubo, Akira Nagasawa, Toshio Nakanishi, Toshiro Takafuta, Junko Tanaka

**Affiliations:** 1 Department of Epidemiology Infectious Disease Control and Prevention, Graduate School of Biomedical and Health Sciences, Hiroshima University, 1-2-3, Kasumi, Minami-ku, Hiroshima, 734-8551 Japan; 2 Hiroshima City Funairi Citizens Hospital, Hiroshima, Japan; 3 Payment Certification Agency (PCA), Ministry of Health, Phnom Penh, Cambodia; 4 Unité de Recherche Clinique de Nanoro (URCN), Institut de Recherche en Science de La Santé (IRSS), Nanoro, Burkina Faso; 5 Department of Gastroenterological and Transplantation Surgery, Hiroshima University, Hiroshima, Japan; 6 Department of Public Health and Health Policy, Graduate School of Biomedical and Health Sciences, Hiroshima University, Hiroshima, Japan; 7 Miyoshi Central Hospital, Hiroshima, Japan

**Keywords:** Infectious diseases, Public health, Epidemiology

## Abstract

This cross-sectional study aimed to investigate the post-acute consequences of COVID-19. We conducted a self-administered questionnaire survey on sequelae, psychological distress (K6), impairments in work performance (WFun), and COVID-19–related experiences of stigma and discrimination in two designated COVID-19 hospitals in Hiroshima Prefecture, Japan, between August 2020 and March 2021. The prevalence of sequelae was calculated by age and COVID-19 severity. Factors independently associated with sequelae or psychological distress were identified using logistic regression analysis. Among 127 patients who had recovered from COVID-19, 52.0% had persistent symptoms at a median of 29 days [IQR 23–128] after COVID-19 onset. Among patients with mild COVID-19, 49.5% had sequelae. The most frequent symptoms were olfactory disorders (15.0%), taste disorders (14.2%), and cough (14.2%). Multivariate analysis showed that age was an independent risk factor for sequelae (adjusted odds ratios [AOR] for ≥ 60 years vs. < 40 years 3.63, p = 0.0165). Possible psychological distress was noted in 30.7% (17.9% of males and 45.0% of females). Female sex and the presence of sequelae were independent risk factors for psychological distress. Of all participants, 29.1% had possible impairments in work performance. Experiences of stigma and discrimination were reported by 43.3% of participants. This study revealed the significant impacts of Long COVID on health in local communities. A large-scale, long-term cohort study is desired.

## Introduction

Coronavirus disease 2019 (COVID-19) is an illness caused by severe acute respiratory syndrome coronavirus 2 (SARS-CoV-2). SARS-CoV-2 infects the host by invading cells via angiotensin-converting enzyme 2 (ACE2)^1^. COVID-19 causes a variety of symptoms, including fever, cough, and olfactory and taste disorders^2^.

In Japan, the Ministry of Health, Labour and Welfare has released guidelines indicating that hospitalized COVID-19 patients can be considered to be no longer harboring SARS-CoV-2 and discharged when (1) 72 h have passed since symptom resolution, and (2) 15 and 10 days have passed since disease onset in patients who did and did not need ventilatory support, respectively^3^. However, a subgroup of patients infected with SARS-CoV-2 experience long-term effects of COVID-19, so-called “long COVID”^4^. Long COVID is a term used to describe the presence of various symptoms persisting weeks or months after infection with SARS-CoV-2, irrespective of the viral strain^5^. The fact that long COVID can considerably reduce the quality of life of infected patients emphasizes the urgent need to identify their effects^6^. Increasing numbers of studies on post-acute and prolonged symptoms of COVID-19 have been published worldwide, helping clarify the long-term course of the disease. However, the types and frequencies of sequelae vary greatly between studies, suggesting differences related to geography, survey methods, and patient background factors^7^.

In this study, patients at two local COVID-19 hospitals were asked to complete a self-administered survey to identify their symptoms, the effects on psychological conditions and work performance, and their experiences of stigma and discrimination related to COVID-19.

## Methods

This cross-sectional study was conducted with the help of two major COVID-19 hospitals (Hospital *A* and *B*) in Hiroshima Prefecture, Japan.

### Study population

Due to the difference in the collaboration agreement between the research team and the two hospitals, the way COVID-19 survivors were invited to participate in the study differed for each hospital. In Hospital *A,* it was based on direct invitations from physicians, and in Hospital *B*, it was based on sending invitation letters.

Hospital *A* is a designated medical institution for Class 2 infectious disease and a regional hub medical center for mild and moderate COVID-19 in its catchment area. Patients who visited Hospital *A* for post–COVID-19 follow-up between September 1, 2020, and March 15, 2021, were asked by their attending physicians to participate in this study. One hundred and eighteen patients gave written consent to participate. Of them, 104 (88.1%) were hospitalized, five (4.2%) stayed at quarantine hotels, and nine (7.6%) stayed at home during their acute infection. Disease severity was mild (i.e. no need for supplemental oxygen) in 93 (78.8%) patients, moderate (i.e. supplemental oxygen needed) in 17 (14.4%) patients, severe (i.e. non-invasive mechanical ventilation use) in five (4.2%) patients, critical (i.e. invasive mechanical ventilation use) in one (0.8%) patient, and unknown in two (1.7%) patients.

Hospital *B* is a designated key medical institution for COVID-19 patients in Hiroshima Prefecture. Forty-three patients who visited this hospital between April 11, 2020, and May 1, 2020, were identified. In August 2020, they were sent a letter asking for participation in this study. Twenty-two patients who visited the outpatient consultation of Hospital *B* for follow-up between August 25, 2020, and September 14, 2020, provided written consent to participate. Of them, 20 (90.9%) were hospitalized, and one (4.5%) stayed at home during the acute infection. The place of medical care during the acute phase was unknown in one (4.5%) case. COVID-19 severity was mild (i.e. no need for supplemental oxygen) in 14 (63,6%) cases, moderate (i.e. supplemental oxygen needed) in one (4.5%) case, and unknown in seven (31.8%) cases.

Overall, 140 post–COVID-19 patients participated in this study.

### Survey methods

Consenting post–COVID-19 patients were asked to complete a set of self-reported anonymous questionnaires. The survey included age, sex, place of medical care, smoking status, medical history, sequelae, screening for impairments in work performance using the Work Functioning Impairment Scale (WFun), screening for mood or anxiety disorders using the 6-item Kessler Psychological Distress Scale (K6), and COVID-19–related experiences of stigma and discrimination.

The WFun questionnaire comprised seven screening questions for measuring impaired work function, and responses were made on a 5-point scale from 1 to 5 (total score range 7–35)^8^. Based on previous findings, scores of 7–13 were classified as normal and those ≥ 14 indicated possible deficits in work performance (14–20, mild; 21–27, moderate; and 28–35, severe)^9^.

The K6 questionnaire consisted of six questions designed to assess the severity of psychological distress on a 5-point scale from 0 to 4 (total score range 0–24)^10^. Based on the *Comprehensive Survey of Living Conditions* conducted by the Ministry of Health, Labour and Welfare of Japan, total scores of 0–4 were categorized as normal, and those ≥ 5 reflected possible mood or anxiety disorders (5–9, mild; 10–14, moderate; and 15–24, severe)^11^.

Severity of COVID-19 and the date of COVID-19 onset were taken from the patients’ medical records. The United States National Institutes of Health defines four levels of disease severity based on the need for respiratory support during hospitalization: no supplemental oxygen, supplemental oxygen, non-invasive mechanical ventilation (non-IMV), and IMV^12^.

### Statistical analysis

Patient characteristics were summarized using medians and interquartile ranges (IQRs) for continuous variables and counts and percentages for categorical variables. The chi-square and Cochran–Armitage trend tests were performed to compare the proportions of patients with sequelae by age group, sex, severity of COVID-19, place of medical care, smoking status, and comorbidities. The chi-square test was also used to compare the prevalence rates of individual sequelae by age group, and to compare the distributions of K6 and WFun total scores by sex and age group.

To identify factors independently associated with sequelae, adjusted odds ratios (AORs) and their 95% confidence intervals (CIs) were estimated using multivariate logistic regression analysis. The response variable was the presence or absence of sequelae. The following explanatory variables were entered into the model simultaneously (forced entry): sex (male or female), age (< 40, 40–59, or ≥ 60 years), disease severity (no supplemental oxygen or ventilatory support needed vs. supplemental oxygen or ventilatory support needed), and smoking status (never smoker vs. current or former smoker). Moreover, the stepwise variable selection method was employed to select additional explanatory variables from among the following comorbidities: hypertension, diabetes mellitus, chronic obstructive pulmonary disease (COPD), malignancy, and cerebrovascular disease (p < 0.25).

Similarly, factors associated with psychological distress were investigated using multivariate logistic regression analysis. The response variable was the K6 total score (cut-off: 5 points). The following explanatory variables were entered into the model simultaneously: sex (male or female), age (< 40, 40–59, or ≥ 60 years), disease severity (no supplemental oxygen or ventilatory support needed vs. supplemental oxygen or ventilatory support needed), smoking status (never smoker vs. current or former smoker), presence of sequelae (yes or no), and COVID-19–related experience of stigma and discrimination (yes or no).

All statistical analyses were performed using JMP® Version 14 (SAS Institute Japan, Ltd., Tokyo) at a significance level of 5%.

This study was approved by the Ethics Committee of Hiroshima University (Approval No. E-2122) and conducted according to the Helsinki declaration. Prior to any study procedures, all patients provided their informed consent.

### Ethics declarations

The Hiroshima University Ethics Committee for Epidemiology approved this study (Approval No. E-2122).

## Results

**Figure 1:**
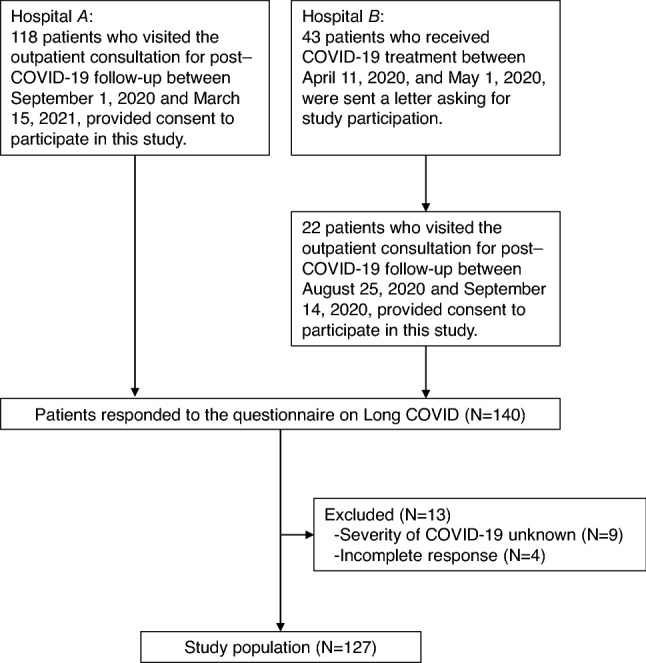
Selection of the study population. The study was conducted among COVID-19 survivors in two designated COVID-19 hospitals in Hiroshima Prefecture, Japan. A total of 127 COVID-19 survivors were included.

One hundred and eighteen patients who visited Hospital *A* for post–COVID-19 follow-up between September 1, 2020, and March 15, 2021, participated in this study and responded to the survey. These patients contracted COVID-19 between April 14, 2020, and February 22, 2021 (during the first to third waves of the COVID-19 pandemic in Japan). During this period, 489 COVID-19 patients were admitted to Hospital *A*, and 118 (24.1%) participated in this study*.* Of 43 patients who visited Hospital *B* for COVID-19 treatment between April 11, 2020, and May 1, 2020 (during the first wave of the COVID-19 pandemic in Japan), 22 (51.2%) responded to the survey between August 25, 2020, and September 14, 2020. Among the 140 patients who responded to the survey, nine had no specific medical records on COVID-19 severity, and four did not fully complete the questionnaires. These 13 patients were excluded, and thus data of 127 patients were analyzed (Fig. 1).

### Patient characteristics

Table 1 summarizes the demographic and clinical characteristics of the study population. The median age was 51 years [IQR 32–58], with ages < 40, 40–59, and ≥ 60 years accounting for 35.4%, 43.3%, and 21.3%, respectively. Males represented 52.8% of the study participants.

**Table 1:**
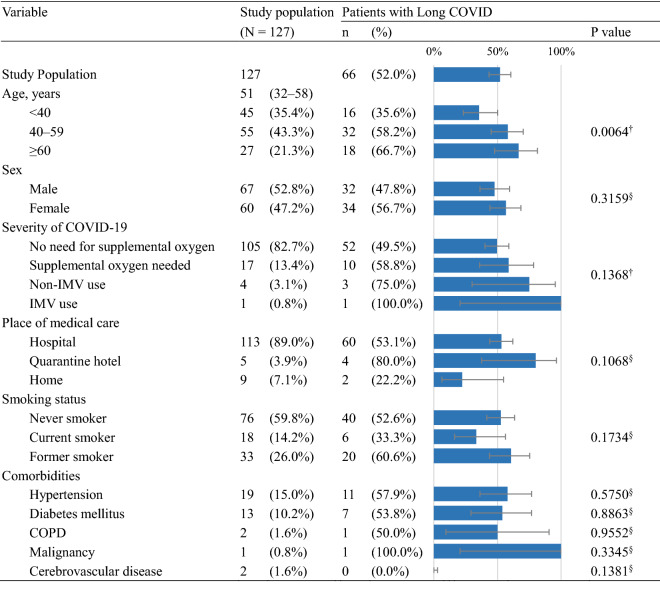
Patient characteristics and prevalence of Long COVID.

Data in the “Study population” column represent the numbers and percentages of relevant patients or median values and their interquartile ranges. Data in the “Patients with Long COVID” section represent the numbers and percentages of patients reporting persistent symptoms at a median of 29 days [IQR 23–128] after COVID-19 onset.

*Non-IMV* non-invasive mechanical ventilation, *IMV* invasive mechanical ventilation, *COPD* chronic obstructive pulmonary disease.

^†^Cochran–Armitage trend test. §Chi-square or Fisher’s exact test.

A large majority (82.7%) of the study population did not need supplemental oxygen, while 13.4% required supplemental oxygen, 3.1% used non-IMV, and 0.8% used IMV. Patients received medical care at hospitals (89.0%), quarantine hotels (3.9%), or their homes (7.1%). Questionnaire responses were collected at a median of 29 days [IQR 23–128] from COVID-19 onset, with a minimum of 11 days and a maximum of 173 days.

### Prevalence rates of sequelae

At the time of the survey, 52.0% (66/127) of the study population reported one or more sequelae (Table 1). Older patients were significantly more likely to have sequelae (p = 0.0064, Cochran–Armitage trend test). The prevalence of sequelae did not significantly differ by sex, severity of COVID-19, place of medical care, smoking status, or comorbidities (chi-square test or Cochran–Armitage trend test).

**Figure 2:**
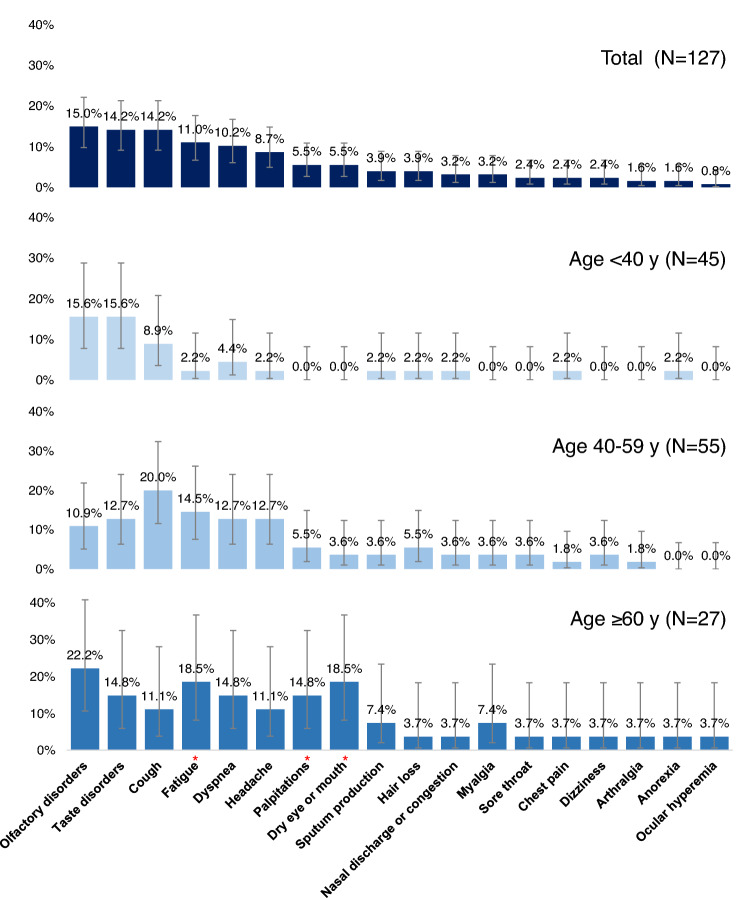
Prevalence of Long COVID symptoms by age. Responses were collected at a median of 29 days [IQR 23–128] after COVID-19 onset. Asterisks (*) indicate statistically significant differences between age groups (p < 0.05, chi-square or Fisher’s exact test).

Specific long COVID symptoms included olfactory disorders (15.0%), taste disorders (14.2%), cough (14.2%), fatigue (11.0%), and dyspnea (10.2%) (Fig. 2). The following symptoms showed age-dependent differences in prevalence: fatigue, palpitations, and dry eye or mouth (p = 0.0401, 0.0327, and 0.0028, respectively), with younger age groups having lower prevalence rates. Other symptoms investigated did not vary by age group.

Multivariate logistic regression analysis showed that the risk of sequelae was significantly higher in older age groups compared with patients < 40 years old (AOR for 40–59 years vs. < 40 years 2.46, 95% CI 1.05–5.73, p = 0.0376; AOR for ≥ 60 years vs. < 40 years 3.63, 95% CI 1.27–10.42, p = 0.0165) (Table 2). Sex, severity of COVID-19, smoking status, and comorbidities were not significantly associated with sequelae.

**Table 2:**
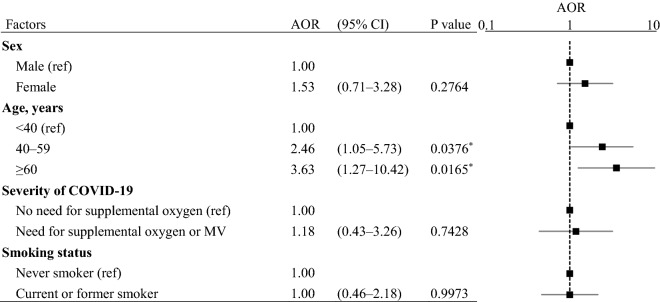
Multivariate analysis results of risk factors for long COVID.

N = 127, R^2^ = 0.0546, Model p = 0.0874.

Data were analyzed using multivariate logistic regression analysis. The response variable was the presence or absence of sequelae. The following explanatory variables were entered into the model simultaneously (forced entry): sex, age, severity of COVID-19, and smoking status. Moreover, the stepwise variable selection method was employed to determine appropriate explanatory variables from among the following comorbidities: hypertension, diabetes mellitus, COPD, malignancy, and cerebrovascular disease. Asterisks (*) indicate statistically significant differences.

*AOR* adjusted odds ratio, *CI* confidence interval, *ref* reference, *MV* mechanical ventilation.

### COVID-19-related experiences of stigma and discrimination

**Figure 3:**
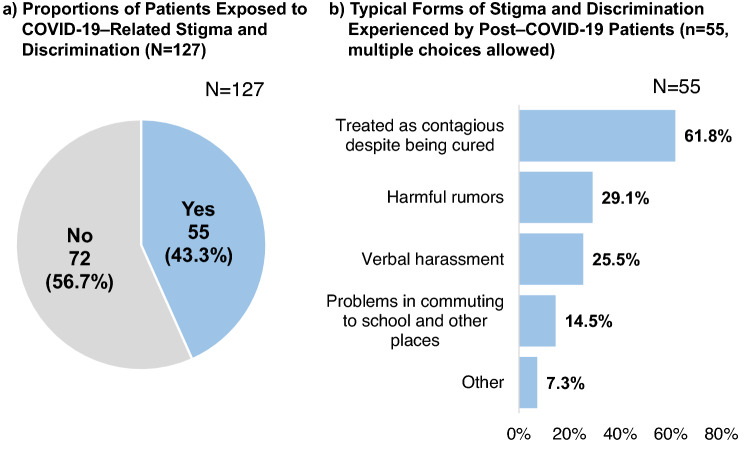
COVID-19–related experiences of stigma and discrimination. The pie chart on the left (**a**) shows the proportion of patients exposed to COVID-19–related stigma and discrimination (n = 127). The bar chart on the right (**b**) presents the typical forms of stigma and discrimination experienced by post–COVID-19 patients (n = 55).

In this study, 43.3% of the study population experienced stigma and discrimination as a result of contracting COVID-19 (Fig. 3). The most common complaints were being treated as contagious despite being cured (61.8%), harmful rumors (29.1%) and verbal harassment (25.5%).

### Impairments in work performance

**Figure 4:**
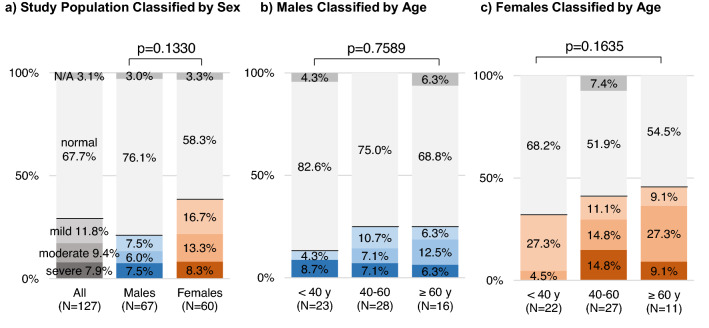
Prevalence rates of deficits in work performance assessed using the WFun questionnaire. The Work Functioning Impairment Scale (WFun) was developed to evaluate deficits in work performance. Total scores of 7–13 were classified as normal, and scores ≥ 14 suggested possible impairments in work performance (14–20, mild; 21–27, moderate; and 28–35, severe). The gray, blue, and orange colors show the distribution of WFun scores for all subjects, males, and females, respectively. The intensity of the color relates to the severity of impairments in work performance. Chi-square test was applied to compare the distributions of WFun scores by sex and age group.

On the WFun questionnaire, 67.7% of the study population had normal scores, whereas 29.1% had possible impairments in work performance (Fig. 4). More specifically, 11.8%, 9.4%, and 7.9% had mild, moderate, and severe impairments, respectively. The frequency distributions of WFun-based impairments were not significantly influenced by sex or age group.

### Psychological distress

**Figure 5:**
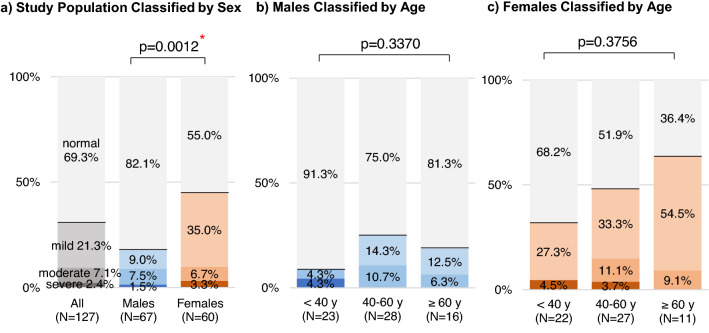
Prevalence of psychological distress assessed using the K6 questionnaire. The 6-item Kessler Psychological Distress Scale (K6) was developed to screen for mood and anxiety disorders. Total scores of 0–4 were classified as normal, and scores ≥ 5 suggested possible mood and anxiety disorders (5–9, mild; 10–14, moderate; and 15–24, severe). The gray, blue, and orange colors show the distribution of K6 scores for all subjects, males, and females, respectively. The intensity of the color relates to the severity of psychological distress. Chi-square test was applied to compare the distributions of K6 scores by sex and age group. Asterisk (*) indicates statistically significant difference (p < 0.05).

On the K6 questionnaire, 69.3% of the study population had normal scores, and 30.7% had possible mood or anxiety disorders (Fig. 5). More specifically, 21.3%, 7.1%, and 2.4% had mild, moderate, and severe disorders, respectively. The risk of psychological distress was higher in females than in males, with 45.0% of females and 17.9% of males having K6 scores ≥ 5 (p = 0.0012, chi-square test). Age did not significantly affect the distribution of patients with mood or anxiety disorders.

Multivariate logistic regression analysis showed that female sex (AOR 3.82, 95% CI 1.51–9.63, p=0.0046) and the presence of sequelae (AOR 4.94, 95% CI 1.91–12.77, p=0.0010) were independent risk factors for psychological distress (Table 3). The risks of psychological distress were typically higher, though not significantly so, in patients who reported stigma and discrimination compared with those who did not (AOR 2.39, 95% CI 0.96–5.91, p=0.0598). Age, severity of COVID-19, and smoking status were not significantly associated with psychological distress.

**Table 3:**
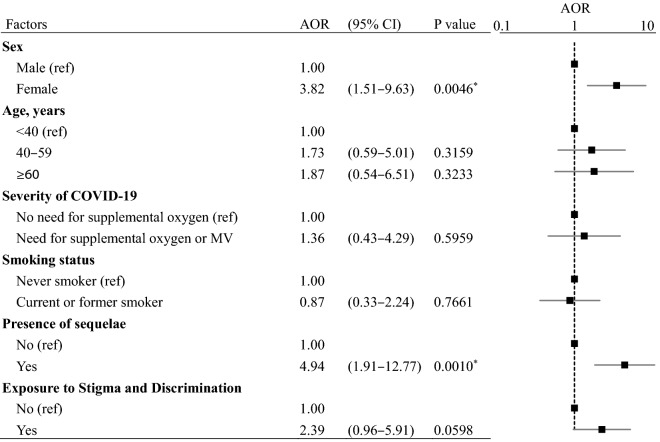
Multivariate analysis results of risk factors for psychological distress (K6 Scores ≥ 5).

N = 127, R^2^ = 0.1932, Model p < 0.0001.

Analyses were conducted using a multivariate logistic regression model. The response variable was the K6 total score (cut-off: 5 points). The following explanatory variables were entered into the model simultaneously (forced entry): sex, age, severity of COVID-19, smoking status, presence of sequelae, and COVID-19–related experience of stigma and discrimination. Asterisks (*) indicate statistically significant differences.

*AOR* adjusted odds ratio, *CI* confidence interval, *ref* reference, *MV* mechanical ventilation.

## Discussion

To investigate the clinical characteristics of Long COVID, we conducted a cross-sectional self-administered questionnaire survey in patients treated at two major COVID-19 hospitals in Hiroshima Prefecture, Japan. Analysis of the responses from 127 recovered patients showed that 52.0% had sequelae at a median of 29 days [IQR 23–128] from COVID-19 onset. The prevalence rates of post-acute or prolonged sequelae of COVID-19 reported in the literature vary considerably depending on the geographic location of the study area and patient background factors^7,13,14^. For instance, the frequency of one or more sequelae in COVID-19 survivors was 32.6% among 488 individuals (2 months post-discharge) in the United States^15^, 87.4% among 143 individuals (2 months post-symptom onset) in Italy^16^, 66% among 150 individuals (2 months post-symptom onset) in France^17^, 74% among 110 individuals (3 months post-symptom onset) in the United Kingdom^18^, 50.9% among 277 individuals (2–3 months post-symptom onset) in Spain^19^, and 76% among 1,733 individuals (6 months post-symptom onset) in China^20^. In a recent Japanese follow-up study of 63 patients who recovered after hospital-based treatment, sequelae were reported in 76% at 14 days, in 48% at two months, and in 27% at four months after COVID-19 onset^21^.

In our study, multivariate logistic regression analysis revealed that older age was an independent and significant risk factor for sequelae. Our findings are consistent with previous research showing that long COVID was more likely with increasing age^22,23^, emphasizing the need for follow-up focusing on long COVID in elderly patients. This study found that sex was not significantly associated with the risk of long COVID, but another report pointed out that long COVID is twice as common in females in males^23^.

Long COVID symptoms are wide-ranging and include fatigue, dyspnea, cough, olfactory disorders, taste disorders and so on. Previous studies reported fatigue as the most common sequelae^6,16,24–26^. In contrast, fatigue was the fourth (11.0%) most frequent sequelae in our study, after olfactory disorders (15.0%), taste disorders (14.2%), and cough (14.2%). COVID-19 affects various tissues and organs, such as those in the respiratory, cardiovascular, and neurological systems. Possible mechanisms underlying long COVID may include immunologic aberrations and inflammatory damage in response to the acute infection, as well as virus-specific pathophysiologic changes^14^. ACE2, the major cell entry receptor for SARS-CoV-2, is extensively expressed in numerous human tissues and organs. Respiratory and other sequelae of long COVID have been found to be common in organs with high ACE2 expression^27,28^. In our study, patients aged ≥ 60 years were more likely than other age groups to report fatigue, palpitations, dry eye or mouth, dyspnea, and sputum production, whereas patients aged < 40 years reported lower prevalence rates of all sequelae except for olfactory and taste disorders. Indeed, the fact that olfactory and taste disorders were more common in younger patients was consistent with a previous report^29^, and may help distinguish symptoms that are more likely to persist in specific age groups.

Even in patients with mild COVID-19 who did not require supplemental oxygen or ventilatory support, sequelae were reported by 49.5% of them. After adjustment for age, sex, and smoking status, COVID-19 severity was not a significant factor associated with sequelae. Previous studies also reported that 53% to 55% of non-hospitalized COVID-19 patients had long COVID symptoms^29,30^. Several reports have pointed out that COVID-19 severity is not associated with sequelae^24,31,32^. These findings suggests that COVID-19 patients should be followed up for persistent symptoms regardless of the severity of COVID-19.

Regarding the impact on occupational performance, 17.4% of the post–COVID-19 patients had moderate or severe impairments (WFun scores ≥ 21). In a previous study that used the WFun questionnaire to investigate degrees of impairment in work performance associated with insomnia, 20% of control subjects without insomnia and 34% of subjects undergoing insomnia treatment had WFun total scores ≥ 21^33^. This simple comparison suggests that post–COVID-19 conditions may influence work performance to only a limited extent.

Long-term psychiatric symptoms after recovery from acute COVID-19 have been reported, including post-traumatic stress disorder (PTSD), depression, anxiety, and obsessive–compulsive symptoms^34–39^. The prevalence of possible mood or anxiety disorders (K6 total scores ≥ 5) were 17.9% for males and 45.0% for females in this study. The Japanese Ministry of Health, Labour and Welfare conducts annually a large-scale comprehensive survey of living conditions of the general population, including the assessment of psychological status by the K6 questionnaire^11,40^. Psychological evaluation in our study used the same tool, allowing for comparison with the general population. In 2019, mood or anxiety disorders prevalence was 24.8% in males and 29.6% in females in the general population^40^. Compared to the data of the general population, mood or anxiety disorders prevalence in our study was lower in male patients and higher in female patients. Moreover, the logistic regression analysis revealed that female sex and the development of sequelae after COVID-19 were independent and significant risk factors for post–COVID-19 mood or anxiety disorders. Previous research also pointed out that female patients with a preexisting psychiatric diagnosis and those managed at home were at a higher risk of psychiatric symptoms after COVID-19^35^. Therefore, female patients and patients with sequelae should receive long-term follow-up with a particular focus on monitoring for mood or anxiety disorders.

Discrimination and stigmatization to healthcare workers, COVID-19 patients, and survivors have been serious challenges across the world during this COVID-19 pandemic^41^. The United Nations Children's Fund (UNICEF), the World Health Organization (WHO), and the International Federation of Red Cross and Red Crescent Societies (IFRC) released a guide to preventing and addressing social stigma, highlighting the importance of communication in combatting COVID-19-related fear and stigma^42^. Overall, 43.3% of our study participants reported exposure to stigma and discrimination related to COVID-19, suggesting that patients may face social challenges as they resume their everyday activities after recovery. Further expansion of the support system for ex-COVID-19 patients by the government and medical institutions and measures to eradicate discrimination are needed.

### Limitations

There are several limitations to this study. First, patient selection may have been biased. At Hospital *A*, outpatient follow-up appointment schedules were based on each patient’s personal preferences, condition at discharge, and imaging findings. Consequently, our patient selection procedure may have skewed the study population towards those who were still symptomatic at discharge. Hospital *B* mailed their patients a letter asking for cooperation in this study, and positive responses were obtained from 51.2% of them. This process may have resulted in an overestimation of the prevalence of long COVID symptoms due to potential selection bias, wherein patients affected by persistent conditions were more likely to participate in this study. Second, the sample size of this study may not be sufficient to conclude about the risks of long COVID. Third, because of its cross-sectional nature, this study was not able to evaluate the duration of individual symptoms. Fourth, the medical institutions of this study were designated for the treatment of patients with mild-to-moderate COVID-19. Consequently, this study did not allow for in-depth analysis of severe cases. Fifth, since the survey was conducted using self-administered questionnaires, it was not possible to make an objective evaluation for each symptom. Despite these limitations, this study identified long COVID in 49.5% of mild cases and demonstrated that COVID-19 may have potentially profound impacts on local communities.

## Conclusion

Long COVID was noted in 52.0% of the study population at a median of 29 days [IQR 23–128] after COVID-19 onset. Common symptoms included olfactory disorders, taste disorders, and cough. The prevalence rates of long COVID varied by age group, with older patients having higher rates. We identified long COVID in half (49.5%) of mild cases and analyzed the potentially profound impacts of long COVID on local communities. Our results warrant a large-scale, long-term cohort study in the future.

## Data Availability

The datasets used and analyzed during the current study are available from the corresponding author on reasonable request.
